# Clinical Epidemiology Characteristics and Antibiotic Resistance Associated with Urinary Tract Infections Caused by *E. coli*

**DOI:** 10.1155/2022/2552990

**Published:** 2022-02-28

**Authors:** Sang Ngoc Nguyen, Huyen Thanh Thi Le, Tam Duc Tran, Lam Tung Vu, Tho Huu Ho

**Affiliations:** ^1^Haiphong University of Medicine and Pharmacy, Haiphong, Vietnam; ^2^Vietnam Military Medical University, Hanoi, Vietnam

## Abstract

**Introduction:**

In individuals with urinary tract infections, *Escherichia coli (E. coli)* is an ubiquitous causative agent and antibiotic resistance is on the rise throughout the world. Therefore, early diagnosis and appropriate choice of antimicrobials are essential. The purpose of our study is to describe some of the clinical and epidemiological characteristics and the laboratory test results of children treated in our hospital for urinary tract infections caused by *E. coli*.

**Methods:**

The study included 128 patients from 2 months to 15 years of age with urinary tract infections caused by *E. coli* and treated at the Haiphong Children's Hospital during the periods of 2011–2013 and 2018–2020.

**Results:**

During the two study periods, 57 and 71 cases, respectively, were included. The most common clinical symptom was fever in 40 and 46 cases, respectively. The proportion of *E. coli*'s resistance to ampicillin increased from 85.3% in 2011–2013 to 97.1% in 2018–2020. In 2011–2013, 70.5% of *E. coli* isolates were resistant to cotrimoxazole, which increased to 81.4% during 2018–2020. During both periods, *E. coli* was highly sensitive to amikacin, at 87% and 95.5%, respectively. In 2018–2020, carbapenems (meropenem and imipenem) and piperacillin were also effective against *E. coli*.

**Conclusion:**

Our study revealed that high fever was the most prevalent clinical characteristic in urinary tract infections caused by *E. coli* in children and *E. coli* was mostly resistant to ampicillin, nalidixic acid, and cotrimoxazole but was highly sensitive to ciprofloxacin, amikacin, piperacillin, meropenem, and imipenem.

## 1. Introduction

Urinary tract infections are common in children [1–3]. Early diagnosis and treatment are necessary for the prevention of complications [4]. *Escherichia coli* (*E. coli*) is a common causative pathogen [5–8], and antibiotics including cotrimoxazole (trimethoprim/sulfamethoxazole), nitrofurantoin, ciprofloxacin, and ampicillin are routinely used for treatment.

Antibiotic resistance is on the rise all around the world, and it remains one of the most pressing issues in the treatment of infectious diseases [9–11]. Similarly, antibiotic resistance is also a critical component in the management of urinary tract infections, particularly those caused by *E. coli* [12, 13]. Authors from several countries have reported an increase in the resistance rate of *E. coli* strains to numerous antibiotics. The capacity of *E. coli* to generate extended beta-lactamase (ESBL) enzyme, which can hydrolyze third-generation cephalosporins and aztreonam but is blocked by clavulanic acid, is partly responsible for its antibiotic resistance [14, 15]. Furthermore, ESBL-producing organisms reveal coresistance to a wide range of antibiotics, limiting therapeutic options [16, 17]. Thus, antimicrobial resistance surveillance is critical for determining its pattern and guiding the selection of empirical therapy.

This study aims to describe some of the clinical characteristics and laboratory test results in children aged from 2 months to 15 years who were diagnosed with the first urinary tract infection caused by *E. coli* in our hospital.

## 2. Materials and Methods

A descriptive cross-sectional study was carried out for two periods (from May 1^st^ 2011 to May 1^st^ 2013 and from December 1^st^ 2018 to December 1^st^ 2020) in the pediatric population in Haiphong City, located in Northern Vietnam, covering about 2 million citizens. All study patients were treated at the Haiphong Children's Hospital, Haiphong, Vietnam.

### 2.1. Inclusion Criteria

A retrospective study was conducted. We chose patients who met the following criteria: patients aged from 2 months to 15 years and diagnosed with first urinary tract infections caused by *E. coli* were included in the study.

Urine specimens were collected from the first UTI and by transurethral catheterization or clean-catch midstream specimen (CCM). A positive culture was defined by the presence of a single organism with the following colony counts: catheterization, >10,000 CFU/mL; CCM, >100,000 CFU/mL [18].

### 2.2. Exclusion Criteria

Patients with age less than two months or those with urinary tract infections not caused by *E. coli* or caused by the combination of *E. coli* and other bacteria or suffering from recurrent UTIs were excluded.

### 2.3. Antibiotic Sensitivity Testing

In 2011–2013, we assessed the sensitivity and resistance of the *E. coli* isolate against 10 antimicrobial agents, including ampicillin, Augmentin, cefotaxime, ceftriaxone, nalidixic acid, gentamicin, amikacin, chloramphenicol, cotrimoxazole, and ciprofloxacin. In 2018–2020, we assessed the sensitivity and resistance of the *E. coli* isolate against 10 antimicrobial agents, including ampicillin, cefotaxime, ciprofloxacin, gentamicin, amikacin, piperacillin, ceftazidime, cefepime, imipenem, and meropenem.

All assessments were determined using the VITEK test method (BioMérieux Vitek2 Compact), which calculates minimum inhibitory concentration (MIC). Susceptibility results are interpreted according to the Clinical Laboratory Standards Institute (CLSI) guidelines [19].

All tests were carried out by the Laboratory Department of Haiphong Children's Hospital.

Multidrug-resistant *E. coli* was defined as nonsusceptible to one agent in at least three antimicrobial categories [20].

### 2.4. Data Analysis

The data were analyzed by Statistical Package for Social Sciences (SPSS) software version 22. To analyze the association between categorical variables, Pearson's chi-square test was used. For a particular drug and organism, antimicrobial susceptibility or resistance rates were estimated as the number of susceptible or resistant organisms divided by the total number of tested organisms.

### 2.5. Ethical Approval

Approval for the study was obtained from the Medical Ethics Council of Haiphong University of Medicine and Pharmacy, and an informed consent was obtained according to the Declaration of Helsinki.

## 3. Results

Our study showed that there was a significant increase in the number of patients diagnosed with first UTIs from 57 cases (in 2011–2013) to 71 cases (in 2018–2020).  Table 1 summarizes the demographic and clinical data of the patients. The number of males was higher than that of females in both periods.

Midstream urine collection and urethral catheterization were the most prevalent techniques for collecting urine samples in both periods (Table 1). Patients with a high fever (>38.5 C) at the time of admission in 2011–2013 accounted for 50.9%, with 29 cases, while those in the period 2018–2020 accounted only for 26.8%, with 19 cases. Moreover, cloudy urine (36.8% in 2011–2013 and 53.5% in 2018–2020)) is the most prevalent reported abnormality. Phimosis was seen in 31.6% of all cases (excluding females and reported as “50% of male cases” rather than all cases).

Table 2 shows that almost all patients had an increase in the white blood cell count and neutrophil count. Both periods witnessed an increase of serum C-reactive protein (≥10 mg/l) in the majority of patients. Leukocyte esterase was detected in almost every patient's urine, with just two children testing negative for the test from 2011 to 2013 (Table 2). In addition, only 10.5% of the children tested positive for nitrite in their urine in 2011–2013, whereas the percentage was 59.2% in the period 2018–2020.

Table 3 illustrates the antibiotics susceptibility study results of *E. coli* strains in 2 study periods. In both research periods, the proportion of ampicillin resistance was the highest, at 85.3% and 97.1% in 2011–2013 and 2018–2020,respectively. Compared between 2011–2013 and 2018–2020, the resistance rates of ampicillin and cotrimoxazole experienced a significant increase to 97.1% and 81.4, respectively, and the same tendency was true for cefotaxime. Our findings also indicated that amikacin, piperacillin, imipenem, and meropenem were still susceptible to *E. coli* isolates, with sensitivity rates of 95.5%, 85.3%, 92.9%, and 91.4%, respectively. There were 8 (14.04%) cases in 2011–2013 and 20 (28.17%) cases in 2018–2020 suffering from multidrug-resistant *E. coli*.

In our study, all patients recovered well after treatment. The average duration of treatment was 11.26 ± 2.33 days.

## 4. Discussion

Urinary tract infections are common in young children, and if not managed appropriately, they can cause permanent renal damage [21, 22]. *E. coli* is the most frequent bacterium that causes UTIs in children [23–25].

According to the findings of this study, urinary tract infection is more frequent among children under the age of 5 years, while the number of patients above 5 years of age was only 3 (5.3%) in 2011–2013 and 13 (18.3%) in 2018–2020. Several authors throughout the world have made similar observations [26, 27]. According to Ismaili [26], the most prevalent age group was children in Belgium under the age of two, which accounted for 75% of the total.

In terms of gender, UTIs usually occur in males more than in females. In addition, in the period from 2011 to 2013, we noted that males outnumber females in the age period from 2 months to under 1 year. Similarly, research of Wu, Chang, and Ismaili also found that males under the age of one had a greater chance of developing urinary tract infections than girls. The results can be explained on the basis of phimosis in males and possibly underlying congenital anomalies, which were examined by ultrasound. Regarding children above the age of one year, girls have more risk of having a urinary tract infection than boys due to factors such as urethral opening close to the anus, short urethra, and high humidity in the perineal environment.

Our study showed that midstream urine collection was the most common urine collection method, followed by urethral catheterization. This distribution was strongly connected to the patient's age because transurethral catheterization was commonly used in patients less than 2 years, while children above 2 years of age used the midstream urine collection method.

In our study, febrile UTI was the most frequent clinical characteristic, accounting for 70.9% of patients, with 50.9% of patients having a high fever at the time of admission. Besides, 96.5% of patients with positive leukocyte esterase in 2011–2013 and 100% of patients with positive leukocyte esterase in 2018–2020 had a positive culture, demonstrating the importance of leukocyte esterase in urinalysis.

Antibiotic resistance in *E. coli*, which causes UTIs, is on the rise all over the world [12, 15]. We noted that there was a remarkable increase in ampicillin resistance to 97.1% from 2011 to 2020. When comparing the current study and the study carried out by Shaki [28] in Southern Israel, ampicillin and cotrimoxazole had the highest rate of *E. coli* resistance. Similarly, in numerous studies throughout the world, such as Guzman's (96.1%), Alanazi's (82.76%), and others, the proportion of *E. coli* resistant to ampicillin is high [10, 12, 28]. Our results also indicate that the resistance rate to *E. coli* of cefotaxime increased from 38.8% in 2011–2013 to 66.7% in 2018–2020. The rise in cephalosporin resistance is a significant limitation for treating children with UTIs [29, 30].

When comparing the current study and the study carried out by Guzman [10] in Venezuela, the combination of amoxicillin and clavulanic acid (Augmentin) had similar results with approximately 25% sensitivity. Our findings indicated that amikacin was still effective for *E. coli* in both periods. We noted that the carbapenem type of antibiotics such as meropenem and imipenem ranked the second highest for *E. coli* sensitivity. Our study showed high sensitivity rates (above 95%) to amikacin, meropenem, and imipenem, which were similar to those reported previously [15]. In terms of multidrug-resistant *E. coli*, our findings revealed a two-fold increase in the number of cases between the two periods, indicating a critical condition of *E. coli* multidrug resistance in UTIs, making it challenging for clinicians to implement a good treatment strategy. In our current practice, we used ciprofloxacin only for systemically unstable adolescents (>13 years old) when *E. coli* is resistant to ampicillin and ceftriaxone, which is consistent with the guidelines of BMJ Best Practice [31].

The main limitation of this research is that the ESBL enzyme of *E. coli* was not determined by polymerase chain reaction (PCR). Other limitations are a smaller sample size, a wide age group, and a single-center study that limits our conclusions' generalizability.

## 5. Conclusions

In summary, high fever was the most prevalent clinical characteristic in urinary tract infections caused by *E. coli* in children. In terms of urinalysis findings, the preponderance of samples revealed the presence of leukocytes in the urine. Amikacin and ciprofloxacin were most effective against *E. coli* and may therefore be used in *E. coli* urinary tract infections. Due to a high prevalence of resistance, ampicillin and cotrimoxazole should be avoided as empirical therapy.

## Figures and Tables

**Table 1:** Demographic and clinical data of the studied children with urinary tract infection caused by *E. coli* (*N* = 128).

Variables	2011–2013 *N* = 57 *n* (%)	2018–2020 *N* = 71 *n* (%)
Age		
2 months–under 1 year	41 (71.9)	24 (33.8)
1–under 5 years	13 (22.8)	34 (47.9)
5–15 years	3 (5.3)	13 (18.3)
Gender		
Male	36 (63.2)	36 (63.2)
Female	21 (36.8)	21 (36.8)
Urine collection		
Urethral catheterization	25 (43.9)	28 (39.4)
Midstream urine collection	32 (56.1)	43 (60.6)
Suprapubic aspiration	0 (0)	0 (0)
Temperature at hospitalization		
<37.5 C	17 (29.8)	25 (35.2)
37.5–38 C	8 (14)	20 (28.2)
>38–38.5 C	3 (5.3)	7 (9.8)
>38.5 C	29 (50.9)	19 (26.8)
Urination disorders		
Cloudy urine	21 (36.8)	38 (53.5)
Hematuria	11 (19.3)	17 (23.9)
Leaky urination	11 (19.3)	24 (33.8)
Painful urination	12 (21.1)	22 (30.9)
Vomiting and diarrhea		
Yes	6 (10.5)	3 (4.2)
No	51 (89.5)	68 (95.8)
Abdominal pain		
Yes	2 (3.5)	5 (7.0)
No	55 (96.5)	66 (93.0)
Urological anomalies	24 (42.1)	15 (21.1)
Phimosis (in males)	18 (31.6)	10 (14.1)
Ureteropelvic junction obstruction	1 (1.75)	2 (2.8)
Kidney stones	4 (7)	4 (5.6)
A duplex kidney	1 (1.75)	0 (0)

**Table 2:** Investigation results of included children (*N* = 128).

Investigation	2011–2013 *N* = 57 *n* (%)	2018–2020 *N* = 71 *n* (%)
White blood cell count		
≥12 × 10^9^/l	36 (63.2)	43 (60.6)
<12 × 10^9^/l	2 (3.5)	28 (39.4)
Neutrophils		
Increase	42 (73.7)	48 (67.6)
Normal	2 (3.5)	10 (14.1)
Serum C-reactive protein (CRP)		
≥10 mg/l	47 (82.5)	56 (78.9)
<10 mg/l	10 (17.5)	15 (21.1)
Leukocyte esterase		
Positive	55 (96.5)	71 (100)
Urine nitrite		
Positive	6 (10.5)	42 (59.2)

**Table 3:** Antibiotic sensitivity test results of *E. coli* isolates (*N* = 128).

Antibiotics	2011–2013				2018–2020			
	*n*	Sensitive	Intermediate	Resistance	*n*	Sensitive	Intermediate	Resistance
		*n* (%)	*n* (%)	*n* (%)		*n* (%)	*n* (%)	*n* (%)
Ampicillin	34	4 (11.8)	1 (2.9)	29 (85.3)	70	2 (2.9)		68 (97.1)
Augmentin	39	11 (28.2)	15 (38.5)	13 (33.3)				
Cefotaxime	49	23 (46.9)	7 (14.3)	19 (38.8)	66	21 (31.8)	1 (1.5)	44 (66.7)
Ceftriaxone	44	21 (47.7)	5 (11.4)	18 (40.9)				
Nalidixic acid	48	22 (45.8)	3 (6.3)	23 (47.9)				
Ciprofloxacin	45	29 (64.4)	11 (24.4)	5 (11.1)	70	46 (65.7)	3 (4.3)	21 (30)
Gentamicin	49	29 (59.2)		20 (40.8)	70	47 (67.1)		23 (32.9)
Amikacin	46	40 (87)	2 (4.3)	4 (8.7)	67	64 (95.5)		3 (4.5)
Cotrimoxazole	44	12 (27.3)	1 (2.3)	31 (70.5)	70	13 (18.6)		57 (81.4)
Chloramphenicol	44	18 (40.9)	9 (20.5)	17 (38.6)				
Piperacillin					68	58 (85.3)	4 (5.9)	6 (8.8)
Ceftazidime					66	23 (31.8)		43 (65.2)
Cefepime					62	25 (40.3)		37 (59.7)
Imipenem					70	65 (92.9)		5 (7.1)
Meropenem					70	64 (91.4)	1 (1.4)	5 (7.1)

## Data Availability

Data and materials used and/or analyzed during the current study are available from the corresponding author on reasonable request.

## References

[1] Giri A., Kafle R., Singh G. K., Niraula N. (2020). Prevalence of E. Coli in urinary tract infection of children aged 1-15 Years in A Medical college of eastern Nepal. *Journal of Nepal Medical Association*.

[2] González M. J., Robino L., Iribarnegaray V., Zunino P., Scavone P. (2017). Effect of different antibiotics on biofilm produced by uropathogenic *Escherichia coli* isolated from children with urinary tract infection. *Pathogens and disease*.

[3] Mattoo T. K., Shaikh N., Nelson C. P. (2021). Contemporary management of urinary tract infection in children. *Pediatrics*.

[4] Pourakbari B., Mamishi S., Shokrollahi M. R. (2019). Molecular characteristics and antibiotic resistance profiles of *Escherichia coli* strains isolated from urinary tract infections in children admitted to children’s referral hospital of Qom, Iran. *Annali di Igiene: Medicina Preventiva e di Comunita*.

[5] Liu Y., Zhang B. L., Wang W. H., Zhang X., Fan S. Y., Li L. (2011). [Antibiotic resistance of pathogens isolated from 181 children with complicated urinary tract infection]. *Zhong Guo Dang Dai Er Ke Za Zhi*.

[6] Robino L., Scavone P., Araujo L. (2014). Intracellular bacteria in the pathogenesis of *Escherichia coli* urinary tract infection in children. *Clinical Infectious Diseases*.

[7] Conover M. S., Hadjifrangiskou M., Palermo J. J., Hibbing M. E., Dodson K. W., Hultgren S. J. (2016). Metabolic requirements of *Escherichia coli* in intracellular bacterial communities during urinary tract infection pathogenesis. *mBio*.

[8] Ganesh R., Shrestha D., Bhattachan B., Rai G. (2019). Epidemiology of urinary tract infection and antimicrobial resistance in a pediatric hospital in Nepal. *BMC Infectious Diseases*.

[9] Luna-Pineda V. M., Ochoa S. A., Cruz-Córdova A. (2018). Features of urinary *Escherichia coli* isolated from children with complicated and uncomplicated urinary tract infections in Mexico. *PLoS One*.

[10] Guzmán M., Salazar E., Cordero V. (2019). Multidrug resistance and risk factors associated with community-acquired urinary tract infections caused by *Escherichia coli* in Venezuela. *Biomedica*.

[11] Goodlet K. J., Benhalima F. Z., Nailor M. D. (2019). A systematic review of single-dose aminoglycoside therapy for urinary tract infection: is it time to resurrect an old strategy?. *Antimicrobial Agents and Chemotherapy*.

[12] Alanazi M. Q., Alqahtani F. Y., Aleanizy F. S. (2018). An evaluation of *E. coli* in urinary tract infection in emergency department at KAMC in Riyadh, Saudi Arabia: retrospective study. *Annals of Clinical Microbiology and Antimicrobials*.

[13] Fan N.-C., Chen H.-H., Chen C.-L. (2014). Rise of community-onset urinary tract infection caused by extended-spectrum *β*-lactamase-producing *Escherichia coli* in children. *Journal of Microbiology, Immunology, and Infection*.

[14] Pana Z. D., Zaoutis T. (2018). Treatment of extended-spectrum beta-lactamase-producing Enterobacteriaceae (ESBLs) infections: what have we learned until now?. *F1000Research*.

[15] Priyadharshana U., Piyasiri L. B., Wijesinghe C. (2019). Prevalence, antibiotic sensitivity pattern and genetic analysis of extended spectrum beta lactamase producing *Escherichia coli* and Klebsiella spp among patients with community acquired urinary tract infection in Galle district, Sri Lanka. *Ceylon Medical Journal*.

[16] Vachvanichsanong P., McNeil E. B., Dissaneewate P. (2020). Extended-spectrum beta-lactamase *Escherichia coli* and *Klebsiella pneumoniae* urinary tract infections. *Epidemiology and Infection*.

[17] Rawat D., Nair D. (2010). Extended-spectrum ß-lactamases in gram negative bacteria. *Journal of Global Infectious Diseases*.

[18] Downs S. M. (1999). Technical report: urinary tract infections in febrile infants and young children. the urinary tract subcommittee of the American academy of pediatrics committee on quality improvement. *Pediatrics*.

[19] Humphries R. M., Abbott A. N., Hindler J. A. (2019). Understanding and addressing CLSI breakpoint revisions: a primer for clinical laboratories. *Journal of Clinical Microbiology*.

[20] Magiorakos A.-P., Srinivasan A., Carey R. B. (2012). Multidrug-resistant, extensively drug-resistant and pandrug-resistant bacteria: an international expert proposal for interim standard definitions for acquired resistance. *Clinical Microbiology and Infection*.

[21] Vollmerhausen T. L., Katouli M. (2014). Molecular characterisation of *Escherichia coli* isolated from hospitalised children and adults with urinary tract infection. *European Journal of Clinical Microbiology & Infectious Diseases*.

[22] Leung A. K. C., Wong A. H. C., Leung A. A. M., Hon K. L. (2019). Urinary tract infection in children. *Recent Patents on Inflammation & Allergy Drug Discovery*.

[23] Vazouras K., Velali K., Tassiou I. (2020). Antibiotic treatment and antimicrobial resistance in children with urinary tract infections. *Journal of Global Antimicrobial Resistance*.

[24] Raman G., McMullan B., Taylor P., Mallitt K.-A., Kennedy S. E. (2018). Multiresistant *E. coli* urine infections in children: a case-control study. *Archives of Disease in Childhood*.

[25] Bradley J. S., Roilides E., Broadhurst H. (2019). Safety and efficacy of ceftazidime-avibactam in the treatment of children ≥3 Months to. *The Pediatric Infectious Disease Journal*.

[26] Ismaili K., Wissing K. M., Lolin K. (2011). Characteristics of first urinary tract infection with fever in children. *The Pediatric Infectious Disease Journal*.

[27] Abdul Raheem R. S., Hussein M. A., Al-Din N. I. (2019). Causative organism of urinary tract infection and drug resistance in children at child’s Central Teaching Hospital in Baghdad City. *Journal of Pakistan Medical Association*.

[28] Shaki D., Hodik G., Elamour S. (2020). Urinary tract infections in children. *European Journal of Clinical Microbiology & Infectious Diseases*.

[29] Madhi F., Jung C., Timsit S. (2018). Febrile urinary-tract infection due to extended-spectrum beta-lactamase-producing Enterobacteriaceae in children: a French prospective multicenter study. *PLoS One*.

[30] Hernández Marco R., Guillén Olmos E., Bretón-Martínez J. R. (2017). Community-acquired febrile urinary tract infection caused by extended-spectrum beta-lactamase-producing bacteria in hospitalised infants. *Enfermedades Infecciosas Y Microbiologia Clinica*.

[31] Santos J. D. *Urinary Tract Infections in Children*.

